# Urine- and Blood-Based Molecular Profiling of Human Prostate Cancer

**DOI:** 10.3389/fonc.2022.759791

**Published:** 2022-03-23

**Authors:** Gang Chen, Guojin Jia, Fan Chao, Feng Xie, Yue Zhang, Chuansheng Hou, Yong Huang, Haoran Tang, Jianjun Yu, Jihong Zhang, Shidong Jia, Guoxiong Xu

**Affiliations:** ^1^ Department of Urology, Jinshan Hospital, Fudan University, Shanghai, China; ^2^ Huidu Shanghai Medical Sciences Ltd, Shanghai, China; ^3^ Research Center for Clinical Research, Jinshan Hospital, Fudan University, Shanghai, China

**Keywords:** biomarker, circulating tumor DNA, liquid biopsy, mutation allele frequency, prostate cancer

## Abstract

**Objective:**

Prostate cancer (PCa) is one of the most common malignant tumors, accounting for 20% of total tumors ranked first in males. PCa is usually asymptomatic at the early stage and the specificity of the current biomarkers for the detection of PCa is low. The present study evaluates circulating tumor DNA (ctDNA) in blood or urine, which can be used as biomarkers of PCa and the combination of these markers may increase the sensitivity and specificity of the detection of PCa.

**Methods:**

Tissue, blood, and urine samples were collected from patients with PCa. All prostate tissue specimens underwent pathological examination. A hybrid-capture-based next-generation sequencing assay was used for plasma and urinary ctDNA profiling. Sequencing data were analyzed by an in-house pipeline for mutation calling. Mutational profiles of PCa and BPH were compared in both plasma and urine samples. Associations of detected mutations and clinical characteristics were statistically analyzed.

**Results:**

A significant association of mutation allele frequencies (MAFs) in the blood samples with patients with metastatic PCa rather than patients with primary PCa, and MAFs are changed after treatment in patients with PCa. Further, the number of mutations in urine is not associated with clinical characteristics of PCa patients, but the frequencies of mutation alleles in the urine are associated with patient age. Comparison of cfDNA aberration profiles between urine and blood reveals more alterations in urine than in blood, including *TP53*, *AR*, *ATM*, *MYC*, and *SPOP* mutations.

**Conclusion:**

This work provides the potential clinical application of urine, in addition to blood, as a powerful and convenient non-invasive approach in personalized medicine for patients with PCa.

## Introduction

Prostate cancer (PCa) is one of the most common malignant tumors in males. The American Cancer Society estimated 191,930 newly diagnosed PCa cases that account for 20% of male tumors ranked first in male cancers and 33,330 deaths ranked second in male cancers in 2020 (1). PCa is usually asymptomatic at the early stage and mostly diagnosed through the blood test of prostate-specific antigen (PSA), a biomarker widely used for over 20 years (2), combined with magnetic resonance imaging (MRI) and digital rectal examination. However, serum PSA is organ-specific rather than cancer-specific, and PSA levels also increase in association with benign prostatic hyperplasia (BPH) and prostatitis. It has been reported that the specificity for the detection of PCa is only about 30% (3). Therefore, alternative biomarkers for the early diagnosis, prevention, and treatment of PCa are eagerly required (4).

Tissue biopsy for the analysis of primary and metastatic lesions is efficient but invasive and is limited by the heterogeneity of individual lesions. As an alternative to a tissue biopsy, the liquid biopsy is minimally invasive and can be easily acquired, thus often being used in clinical practice when a tumor sample is unavailable or difficult to obtain. The detection of circulating nucleic acids in human plasma was first described in 1948 (5). In recent years, liquid biopsy has achieved clinical utility for predicting the responsiveness of treatment, drug resistance, and disease recurrence through analyzing circulating tumor DNA (ctDNA) in blood samples. Generally, tumor cells are constantly shed in the patient’s body, releasing cellular components such as DNA and proteins, which may enter into the blood circulation. Therefore, the peripheral blood from tumor patients may contain ctDNA, extracellular vesicles, and circulating tumor cells (CTCs) carrying tumor genomic information which might reflect tumor burden and progression (6).

A previous study showed that primary tissue and ctDNA share relevant somatic alterations, suggesting that ctDNA can be used for molecular subtyping in metastatic castration-sensitive PCa (7). Moreover, it has been reported that a ctDNA assay is sufficient to identify all driver DNA alterations presented in matched tissue in most metastatic castration-resistant PCa (mCRPC) cases, indicating that the management of patients with mCRPC could be based on ctDNA profiling alone (8). In general practice, liquid biopsy analysis can guide the use of androgen receptor (AR)-targeted therapy (9). Most interestingly, DNA fragments can also be detected in urine. The detection of the Y-chromosome SRY gene fragment in the urine supernatant was reported in 1999 (10), indicating the potential use of urine-based DNA biomarkers. Ten years later, transrenal DNA (tr-DNA) filtered by the kidney from the blood was detected in the urine and confirmed to be cell-free DNA (cfDNA) (11). Urine is also an effective source of tumor DNA and is more patient-friendly due to its non-invasive collection methods (12).

Liquid biopsy has been increasingly used in clinical applications. However, the diagnostic value of blood-based ctDNA and urinary ctDNA has not been fully validated. In the present study, we determined whether these circulating nucleic acids could be used as biomarkers of PCa and if the combination of these markers could increase the sensitivity and specificity of the early detection of PCa.

## Materials and Methods

### Patient and Study Design

A total of 54 plasma samples and 20 urine samples were collected from patients with PCa (33 cases) and BPH (15 cases) at Jinshan Hospital, Fudan University from March 2017 to November 2018. The follow-up of patients was from March 2017 to December 2020. Written informed consent was obtained from each participant. The study was approved by the Ethics Committee of Jinshan Hospital (approval # IEC-2020-S27).

### Tissue Sample Preparation and Pathological Assessment

The clinical diagnosis was based on the PSA level, a transrectal needle biopsy of the prostate gland, and histopathological examination. After surgery or biopsy, tissues from patients without neoadjuvant therapies such as hormonal therapy, chemotherapy, or radiotherapy were immediately frozen in liquid nitrogen and stored at -80°C for subsequent use. Fresh normal, BPH, and PCa tissues were used in this study. All tissues from patients without neoadjuvant therapies such as hormonal therapy, chemotherapy, or radiotherapy were immediately frozen in liquid nitrogen after surgery or biopsy and stored at -80°C for subsequent use.

All prostate tissue specimens underwent pathological examination after surgery in the Department of Pathology, Jinshan Hospital. The clinical diagnosis including histological grade and TNM stage were made by experienced pathologists and urologists according to the World Health Organization (WHO) classification and the American Joint Committee on Cancer (AJCC) Manual (eighth edition).

### Liquid Sample Preparation and Cell-Free DNA Extraction

Peripheral blood was collected in EDTA vacutainer tubes and processed within 2 hours. The blood samples were allowed to clot for 30 min before centrifugation for 15 minutes at 1000 x *g* and the plasma was collected and stored at -80°C prior to cfDNA extraction. For urine sample collection, an in-house urine collection kit was developed to maintain the integrity of urinary cfDNA and to facilitate the transportation of urine samples. Morning urine was obtained through the urine collection cup and transferred into four vacuum tubes, where the urine samples were mixed thoroughly with prefilled preservation buffer.

### Next-Generation Sequencing (NGS)-Based Liquid Biopsy

ctDNA sequencing and bioinformatic analysis were conducted based on previously published methods (13, 14). Briefly, plasma and urinary cfDNAs were extracted using the QIAamp circulating nucleic acid kit (Qiagen) from plasma and urine samples, respectively. Up to 30 ng of extracted cfDNA were used for library construction and then the amplified libraries were subjected to hydrid-based target panel (PredicineCARE) capture. The library was loaded to an Illumina HiSeqX Ten for 2 x 150 bp pair-end sequencing. Lastly, the sequencing data were analyzed by an in-house developed pipeline to identify point mutation, insertions, or deletions. For plasma samples, mutations with allele frequencies greater than 0.1% and with at least 4 unique supporting reads were called. For urine samples, mutations with allele frequencies greater than 0.5% and with at least 4 unique supporting reads were called.

### MSK-IMPACT Dataset

Publicly available prostate cancer mutation data were downloaded from MSK-IMPACT (15), in which 504 prostate cancer patients were reported in this study.

### Blood Tests for PSA, Hemoglobin, Creatin, Albumin, Glucose

Serum PSA levels were tested using the Access Hybritech PSA kit (Beckman Coulter, Brea, CA, USA). The normal concentration of PSA ranged from 0 to 4 ng/mL. Hemoglobin levels were determined using the sodium lauryl sulfate – hemoglobin (SLS-Hb) method. Creatin levels were tested using the enzyme-linked immunosorbent assay (ELISA). Albumin levels were tested using bromocresol green. Glucose levels were tested using hexokinase ultraviolet colorimetry.

### Microscopic Hematuria

Microscopic hematuria was diagnosed when there was no obvious change in the appearance of urine, but following centrifugation, there were more than 3 red blood cells per high-power field of view of the pelleted cell sample during microscopic examination.

### Computed Tomography (CT) Scanning

CT scans were conducted using a 64-detector row scanner (Brilliance, Philips, Cleveland, OH, USA). The thickness of a section was 1 mm.

### Statistical Analysis

Statistical analyses were conducted using the R language software (version 4.0) (https://www.r-project.org/) and Prism 8 (GraphPad Software, San Diego, CA, USA). Differences in gene mutation allele frequency (MAF) between patient groups were detected using a Mann-Whitney U test. The association of MAF or the number of mutations and categorized clinicopathological characteristics of the PCa patients was analyzed using a Mann-Whitney U test for two categories and a Kruskal-Wallis test for categories greater than two. The Association of MAF and continuous clinical features were evaluated by Spearman’s correlation. Differences in the number of mutations between PCa and BPH patients were detected using a Mann-Whitney U test. A *P*-value of less than 0.05 was considered statistically significant.

## Results

### PCa Patient Clinicopathological Characteristics

A total of 33 PCa patients and 15 BPH patients were enrolled in the present study. Compared to BPH, PCa was positively correlated with age and PSA concentration. We found that patients with PCa were older (*P* = 0.016) and the level of PSA was higher (*P* = 0.002) (**Supplementary Table 1**). Next, we compared the number of variants (< 2 *vs.* ≥ 2) among PCa patients. 19 PCa patients had variants < 2 and 14 PCa patients had variants ≥ 2. PCa patients with variants ≥ 2 were more advanced for M stage (*P* = 0.035) (**Table 1**).

**Table 1:** Correlation between No. of mutations and clinicopathological characteristics of the PCa patients.

Characteristic	No. of variants< 2 (n=19)	No. of variants≥ 2 (n=14)	*P*-value
Age at diagnosis, mean ± SD	77.14 ± 6.70	78.00 ± 6.62	0.7249
PSA, median (range)	56.0 (5.04-3327.0)	103.1 (10.37-6132.0)	0.4160
History of radiotherapy			0.8230
Yes, n (%)	1 (5.3)	1 (7.1)	
No, n (%)	18 (94.7)	13 (92.9)	
Castration-resistant			0.4791
Yes, n (%)	2 (10.5)	1 (7.1)	
No, n (%)	17 (89.5)	12 (85.7)	
Unknown, n (%)	0 (0.0)	1 (7.1)	
Grade group, n (%)			0.9136
1, n (%)	2 (10.5)	1 (7.1)	
2, n (%)	2 (10.5)	2 (14.3)	
3, n (%)	2 (10.5)	2 (14.3)	
4, n (%)	2 (10.5)	3 (21.4)	
5, n (%)	6 (31.6)	4 (28.6)	
Unknown, n (%)	5 (26.3)	2 (14.3)	
T stage, n (%)			0.6693
cT1, n (%)	6 (31.6)	2 (14.3)	
cT2, n (%)	7 (36.8)	5 (35.7)	
cT3, n (%)	2 (10.5)	3 (21.4)	
cT4, n (%)	3 (15.8)	2 (14.3)	
cTx, n (%)	1 (5.3)	2 (14.3)	
N stage, n (%)			0.9162
N0, n (%)	12 (63.2)	9 (64.3)	
N1, n (%)	2 (10.5)	2 (14.3)	
Nx, n (%)	5 (26.3)	3 (21.4)	
M stage, n (%)			**0.0346**
M0, n (%)	16 (84.2)	7 (50.0)	
M1, n (%)	3 (15.8)	7 (50.0)	
Stage group, n (%)			0.3035
I, n (%)	1 (5.3)	0 (0.0)	
II, n (%)	2 (10.5)	2 (14.3)	
III, n (%)	8 (42.1)	2 (14.3)	
IV, n (%)	5 (26.3)	8 (57.1)	
Unknown, n (%)	3 (15.8)	2 (14.3)	
Treatment naïve, n (%)			0.3174
Yes, n (%)	7 (36.8)	7 (50.0)	
No, n (%)	12 (63.2)	6 (42.9)	
Unknown, n (%)	0 (0.0)	1 (7.1)	

Contingency tables were analyzed using the chi-square test. Numerous data chosen from the normal population were analyzed using Student’s t-test. Numerous data that were not chosen from the normal population were analyzed using the Mann-Whitney test. The bold value indicates a statistical significance. T, primary tumor; N, regional lymph node metastasis; M, distant metastasis.

### Mutation Allele Frequencies Are Significantly Higher in The Plasma of Patients With PCa and Are Related to Metastasis

**Figure 1:**
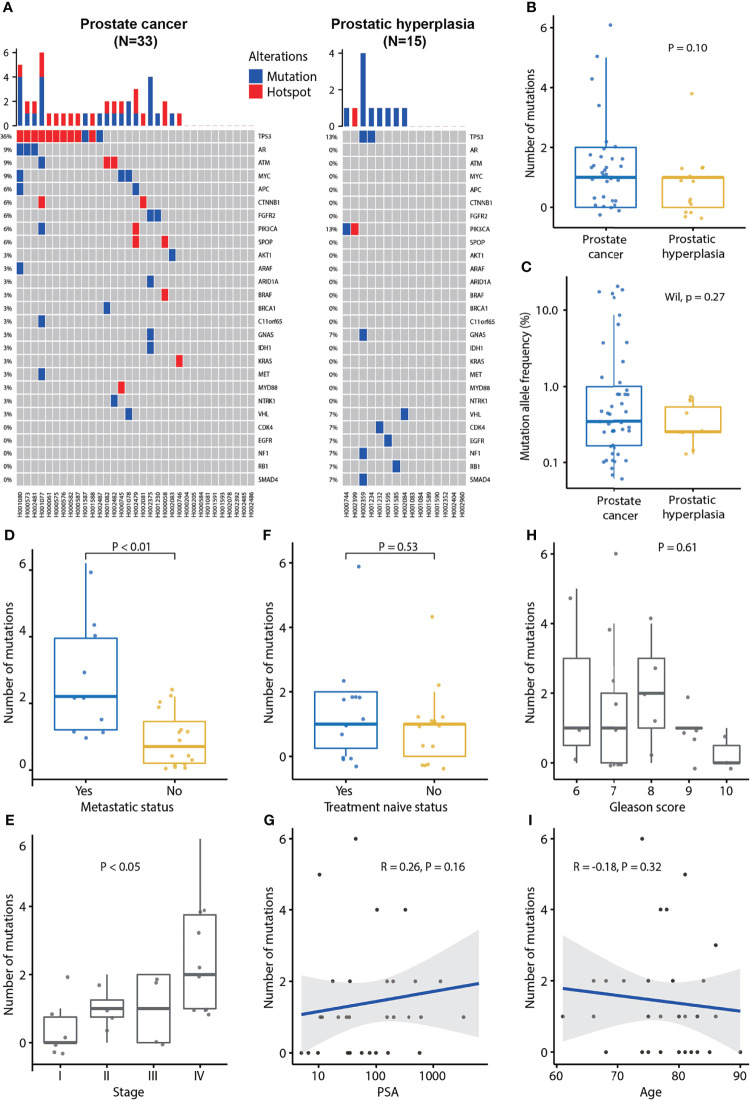
Plasma cfDNA mutation profiles and association of the number of mutations detected in plasma cfDNA and clinical characteristics of prostate cancer. **(A)** cfDNA mutation profile of prostate cancer and prostatic hyperplasia. Blue color indicates mutation and red color indicates hotspot mutation which is defined as a mutation with occurrence greater than 20 in the COSMIC database. **(B)** The number of mutations in prostate cancer and prostatic hyperplasia. **(C)** Mutation allele frequencies in prostate cancer and hyperplasia. **(D)** Association of the number of mutation and metastatic status. p-value was calculated by the Mann-Whitney U test. **(E)** Association of the number of mutations and tumor stage. P-value was calculated by the Kruskal-Wallis test. **(F)** Association of the number of mutations and treatment naïve status. P-value was calculated by the Mann-Whitney U test. **(G)** Association of the number of mutations and PSA. Spearman’s rank correlation coefficient and the corresponding P-value are shown. **(H)** Association of the number of mutations and Gleason score. P-value was calculated by the Kruskal-Wallis test. **(I)** Association of the number of mutations and age. Spearman’s rank correlation coefficient and the corresponding p-value are shown. Each dot indicates one sample.

Next, we compared MAFs between PCa and BPH in plasma DNA sequencing. We observed a trend that patients with PCa have higher MAFs detected (**Figures 1A, B**), although it does not reach statistical significance (*P* = 0.10). Further, statistical analysis showed that there was no difference in MAF (*P* = 0.27; **Figure 1C**) between PCa and BPH.

Further analysis demonstrated that the MAF was associated with metastasis status. MAFs were significantly higher in metastatic PCa (*P* = 0.02) and lower in treatment naïve PCa patients (*P* = 0.03; **Supplementary Figures 1A, B**). MAF was not associated with Gleason score (*P* = 0.40), tumor stage (*P* = 0.17), PSA (*P* = 0.18), or age (*P* = 0.32) (**Supplementary Figures 1C-F**). Patients with PCa at stage IV tended to have higher MAFs (**Supplementary Figure 1D**).

Furthermore, we found that the number of mutations was associated with metastatic status and tumor stage. The number of mutations was significantly higher in metastatic PCa (*P* < 0.01; **Figure 1D**). Furthermore, the number of mutations was significantly associated with the tumor stage (*P* < 0.05; **Figure 1E**), being the highest frequent in patients with PCa at stage IV. However, the number of mutations was not associated with treatment naïve status (*P* = 0.53), PSA (*P* = 0.16), Gleason score (*P* = 0.61), or age (*P* = 0.32; **Figures 1F–I**).

### PSA Levels Are Not Associated With Metastatic Status

**Figure 2:**
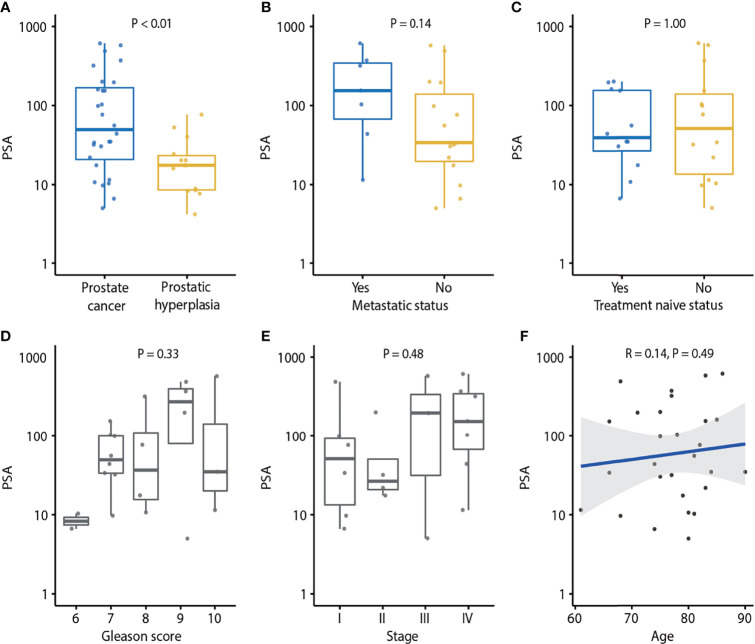
Association of PSA and clinical characteristics of prostate cancer. **(A)** PSA in prostate cancer and prostatic hyperplasia. P-value was calculated by the Mann-Whitney U test. **(B)** Association of PSA and metastatic status. P-value was calculated by the Mann-Whitney U test. **(C)** Association of PSA and treatment naïve status. p-value was calculated by Mann-Whitney U test. **(D)** Association of PSA and Gleason score. P-value was calculated by the Kruskal-Wallis test. **(E)** Association of PSA and tumor stage. P-value was calculated by the Kruskal-Wallis test. **(F)** Association of PSA and age. Spearman’s rank correlation coefficient and the corresponding P-value are shown. Each dot indicates one sample.

We found that PCa patients had higher plasma PSA levels compared to BPH patients (**Figure 2A**). The serum PSA was significantly higher in PCa than in BPH patients (*P* < 0.01). However, there was no association of PSA concentration with MAF (P = 0.18; **Supplementary Figure 1E**), number of variants (*P* = 0.16; **Figure 1G**), metastatic status (*P* = 0.14; **Figure 2B**), treatment naïve status (*P* = 1.00; **Figure 2C**), Gleason score (*P* = 0.33; **Figure 2D**), tumor stage (*P* = 0.48; **Figure 2E**), or age (*P* = 0.49; **Figure 2F**).

### Plasma cfDNA Genomic Alterations Across Samples

**Figure 3:**
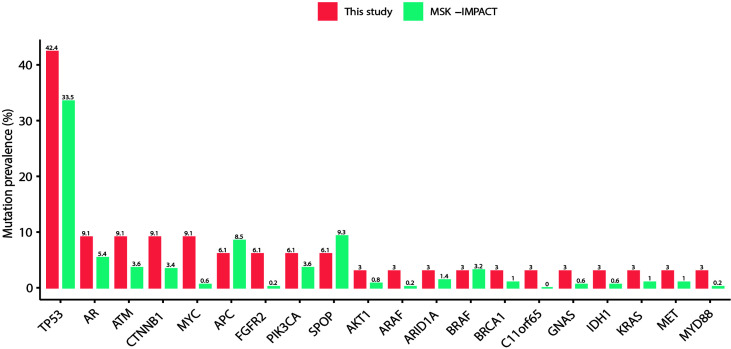
Detection of gene mutation prevalence in plasma cfDNA samples. The plasma spectrum of alterations in this study was almost identical and correlated with tissue samples in the MSK-IMPACT Clinical Sequence Cohort of prostate cancer (37 vs. 504 samples).

Alterations in some genes were detected in PCa patients, including *TP53*, *AR*, *ATM*, *MYC*, *APC*, *CTNNB1*, and *SPOP*, etc. (**Figures 1A**, **3**). In BPH, several genes were altered, including *TP53*, *PIK3CA*, *GNAS*, *VHL*, *CDK4*, *EGFR*, *NF1*, *RB1*, and *SMAD4* (**Figure 1A**). One patient harbored a *PIK3CA* p.Arg108His hotspot mutation. The spectrum of alterations in our study was almost identical and correlated with the clinical sequence cohort in the MSK-IMPACT database (37 *vs.* 504 samples; **Figure 3**). The mutation prevalence of SPOP and APC is lower in our dataset, which may be due to the limited sample size.

### Mutation Allele Frequencies Are Changed After Treatment in Patients With PCa

**Figure 4:**
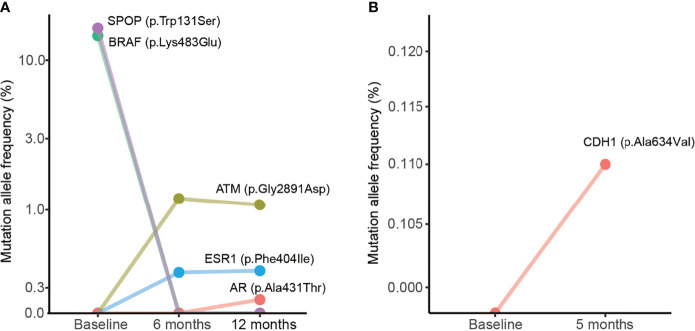
Dynamic changes of mutation allele frequencies in plasma samples during treatment. **(A)** Plasma samples from the case #1 patient. **(B)** Plasma samples from the case #2 patient. The baseline indicates plasma samples collected from the patient before treatment.

Dynamic changes in MAFs in plasma samples during treatment were observed. For instance, in the case #1 patient, we observed changes of MAFs in *SPOP*, *BRAF*, *ATM*, *ESR1*, and *AR* after 6 or 12 months of treatment (**Figure 4A**). This patient was a 66-year-old man whose prostate biopsy revealed prostate adenocarcinoma with a Gleason score of 7 (3 in major + 4 in minor). At the time of diagnosis, the CT scan did not show bone metastasis (**Supplementary Figure 2A**), and the liquid biopsy revealed *BRAF* and *SPOP* mutations (**Figure 4A**). After oral treatment with bicalutamide and leuprolide for 4 months, the CT scan indicated multiple bone metastasis (**Supplementary Figure 2A**). After continuous treatment for 6 months, the liquid biopsy revealed the presence of *ATM* and *ESR1* mutations and the disappearance of *BRAF* and *SPOP* mutations. After treatment for 12 months, the liquid biopsy revealed *ATM*, *ESR1*, and *AR* mutations (**Figure 4A**). After treatment for 41 months with follow-up to August 2020, a CT scan still indicated multiple bone metastases (**Supplementary Figure 2A**). The case #2 patient was an 83-year-old man whose prostate biopsy revealed prostate adenocarcinoma with a Gleason score of 10 (5 in major + 5 in minor). At the time of diagnosis, the CT scan did not show metastasis (**Supplementary Figure 2B**) and the liquid biopsy did not reveal any gene mutations (**Figure 4B**). Subsequently, case #2 patient underwent bilateral orchiectomy and received oral bicalutamide treatment. After treatment for 5 months, the liquid biopsy revealed a *CDH1* mutation (**Figure 4B**). After treatment for 25 months and follow-up to April 2019, CT scans indicated cancer recurrence and pelvic lymph node metastases (**Supplementary Figure 2B**). These data suggest that dynamic changes of MAFs may relate to metastases.

### Number of Mutations and Mutation Allele Frequencies Detected in Urine From PCa Patients

**Figure 5:**
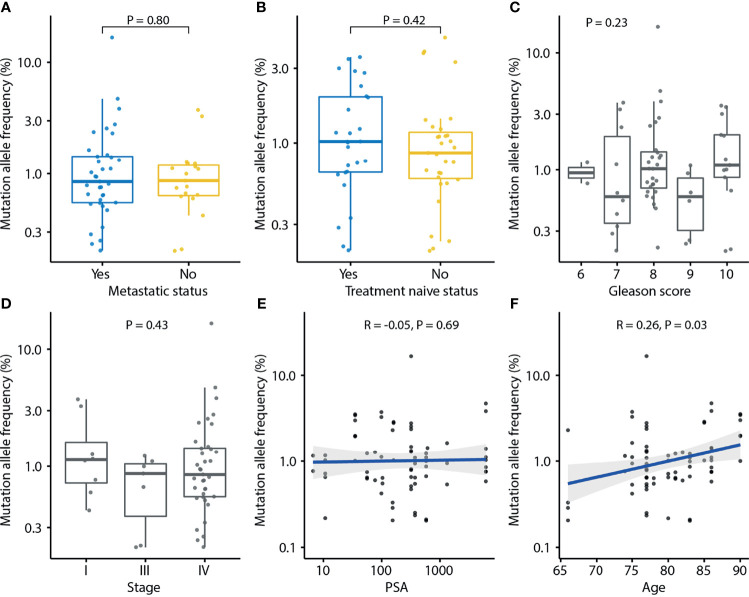
Association of mutation allele frequency (MAF) detected in urine cfDNA and clinical characteristics of prostate cancer. Each dot indicates one sample. **(A)** Association of MAF and metastasis status. P-value was calculated by the Mann-Whitney U test. **(B)** Association of MAF and treatment naïve status. P-value was calculated by the Mann-Whitney U test. **(C)** Association of MAF and Gleason score. P-value was calculated by the Kruskal-Wallis test. **(D)** Association of MAF and tumor stage. P-value was calculated by the Kruskal-Wallis test. **(E)** Association of MAF and PSA. Spearman’s rank correlation coefficient and the corresponding P-value are shown. **(F)** Association of MAF and age. Spearman’s rank correlation coefficient and the corresponding P-value are shown. Each dot indicates one sample.

In the urinary cfDNAs study, the number of mutations and the frequency of mutation alleles tended to be higher in PCa than in BPH (*P* = 0.25 and *P* = 0.06; **Supplementary Figures 3A, B**), indicating that urine may represent an alternative source for the diagnosis of PCa. The number of mutations in urine was not associated with clinical features such as metastatic status (*P* = 0.74). treatment naïve status (*P* = 0.95), Gleason score (*P* = 0.31), stage (*P* = 0.39), PSA level (*P* = 0.44), or age (*P* = 0.66) (**Supplementary Figure 4**). MAF was positively correlated with age (*P* = 0.03) but not associated with metastatic status (*P* = 0.80), treatment naïve status (*P* = 0.42), Gleason score (*P* = 0.23), tumor stage (*P* = 0.43), or PSA level (*P* = 0.69) (**Figure 5**).

### cfDNA Genomic Alterations Across Urine Samples and Matched MSK-IMPACT

There were several altered genes in urine samples (n = 15) in our detection that were almost identical and correlated with the clinical sequence cohort (n = 504 samples) in the MSK-IMPACT database (**Supplementary Figure 5**).

### Comparison of cfDNA Between Urine and Plasma

**Figure 6:**
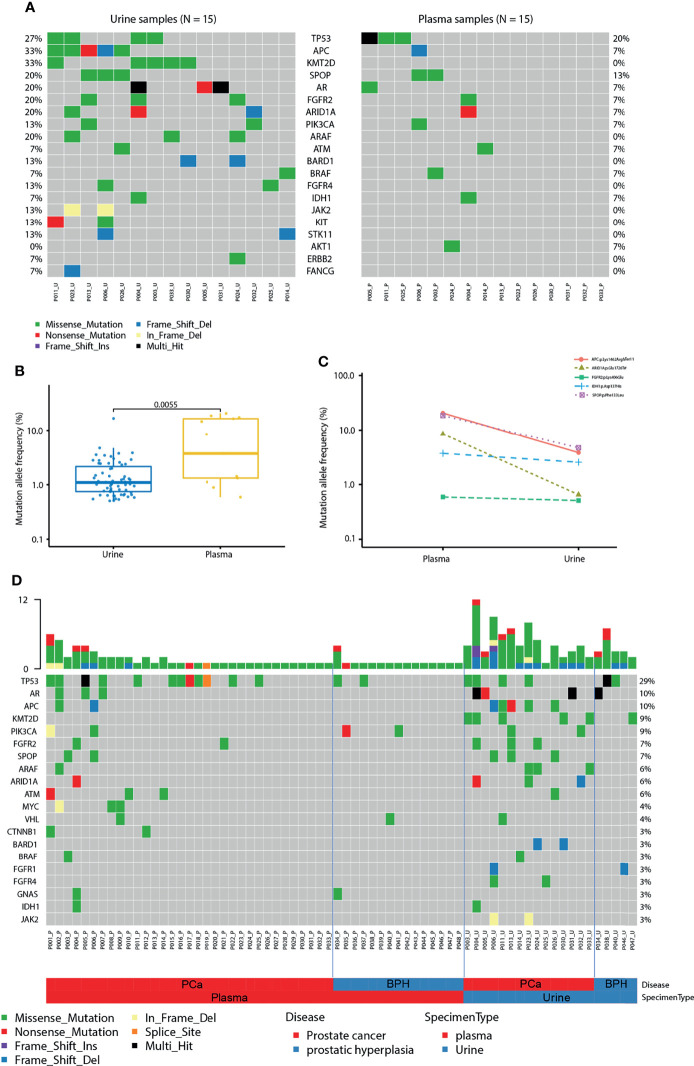
Comparison of cfDNA between urine and plasma. **(A)** Mutation landscape of urine (left) and plasma (right) samples from patients with prostate cancer. Some genes with only one mutation were not shown in the figure. **(B)** Mutation allele frequencies detected in paired urine and plasma samples from patients with prostate cancer. **(C)** Mutation allele frequencies of matched mutations detected in paired urine and plasma samples. **(D)** The landscape of mutations in urine and plasma samples from patients with prostate cancer (PCa) and benign prostatic hyperplasia (BPH).

Next, we compared the sequencing results using urinary cfDNA to plasma cfDNA. 15 patients with paired urine and plasma samples were compared. Interestingly, the mutation profiles in plasma and urine are largely different. The prevalence rates are higher in urine than it in plasma, including *TP53* (27% *vs.* 20%), *APC* (33% *vs.* 7%), *KMT2D* (33% *vs.* 0%), *SPOP* (20% *vs.* 13%), *AR* (20% *vs.* 7%), *FGFR2* (20% *vs.* 7%), *ARID1A* (20% *vs.* 7%), *PIK3CA* (13% *vs.* 7%) and *ARAF* (20% *vs.* 0%) (**Figure 6A**). The average mutations per sample were higher in urine than it in plasma (5.2 *vs.* 1.3, *P* = 0.002**)**. Interestingly, the MAFs were detected to be significantly higher in plasma than in urine by using the same variant calling cut-off 0.5% (*P* < 0.01; **Figures 6B, C**). From the comparison of the mutation landscape, we detected more mutations in PCa samples than BPH samples as well as more mutations in urine samples than plasma samples (**Figure 6D**).

## Discussion

The mechanism underlying the shift from an indolent castration-sensitive phenotype to a lethal castration-resistant PCa (CRPC) is not clear. Patients with PCa, compared with patients with BPH, had more genetic mutations in *TP53*, *AR*, *ATM*, *MYC*, *ESR1*, and *SPOP* genes and most of them were hotspot mutations. Mutations were also found in BPH samples which, as a group, harbored more *NF1*, *RB1*, and *SMAD4* mutations. In addition, *TP53*, *ATM*, *SPOP*, and *AR* gene mutations were more common in metastatic patients, which was consistent with the previous studies (16).

The *TP53* gene is a tumor suppressor gene and its inactivation plays an important role in tumorigenesis. The present study detected *TP53* mutation in the plasma and urine of patients with PCa. Indeed, *TP53* is the most frequently mutated gene in human cancers, and its mutations can cause cell cycle disorders, leading to abnormal proliferation and malignant transformation (17). About 3-47% of PCa specimens have *TP53* mutations and 2-15% contain homozygous deletions (18, 19). Previous studies have shown that a TP53R270H mutation was sufficient to induce PCa in mice (20). Mutations or deletions of *TP53* are also associated with an increased risk of the recurrence of PCa (21). The inactivation of p53 protein, encoded by the *TP53* gene, in the primary PCa may be predictive of inferior outcomes in response to novel hormonal therapies in CRPC (22).

The current study also detected mutations in the *AR* gene, which functions as a steroid hormone-activated transcription factor. *AR* gene aberrations are rare in prostate cancer before primary hormone treatment but emerge with castration resistance. Several studies reported the association between *AR* copy number gain in serum and abiraterone resistance (23–25). RNA in urine-derived extracellular vesicles is available for androgen receptor splice variant 7 (AR-V7) expression analysis, which is higher in patients with advanced PCa (26). Thus, liquid biopsy has detected *AR* gene mutations during late-stage PCa and in association with resistance to androgen deprivation therapy. We observed AR variants in 3/33 PCa patients.

The *ATM* gene is a DNA-damage response gene that is commonly mutated in cancer. The mutation status of *ATM* distinguishes lethal *vs.* indolent PCa and is associated with earlier age at death and shorter survival time (27). In addition, the mutation status of *ATM* is associated with grade reclassification among men undergoing active surveillance (28). Therefore, detecting the mutation status of *ATM* by liquid biopsy may aid decision-making for PCa screening and treatment.

The *MYC* gene is a proto-oncogene and encodes a nuclear phosphoprotein that plays a role in cell cycle progression, apoptosis, and cellular transformation. The *MYC* gene is overexpressed and contributes to the tumorigenesis and progression of PCa (29). A previous study reported that amplification of *MYC* ctDNA in serum was associated with worse failure-free survival and/or overall survival (OS), which remained significant after multivariable analysis (30).

*SPOP* gene mutation is common in solid tumors, especially in PCa (31–33). T speckle-type POZ protein, encoded by the *SPOP* gene, is a substrate adaptor of the cullin3-RING ubiquitin ligase and localizes to nuclear speckles. Recent genomic studies reported a decreased frequency of *SPOP* mutations in mCRPC when compared to localized disease (16, 34). Detection of *SPOP* mutations in serum is expected to become a new biomarker for PCa.

In the current study, liquid biopsy profiling of the case #1 patient detected *BRAF* and *SPOP* mutations after the pathological diagnosis and before treatment. Following androgen deprivation treatment, the liquid biopsy revealed the presence of *ATM*, *ESR1*, and *AR* mutations, but not *BRAF* or *SPOP* mutations. Previous studies discovered that *ESR1* mutation was associated with bone metastasis of breast cancer (35, 36). However, the role of *ESR1* mutation in PCa has not been investigated. The *CDH1* gene encodes E-cadherin protein, which mediates calcium cell-cell adhesion. *CDH1* mutations are associated with metastatic progression in various malignant tumors (37–40). Our liquid biopsy detected a *CDH1* mutation in the case #2 patient after 7 months of treatment before the CT scan, which revealed cancer recurrence and pelvic lymph node metastases, indicating that liquid biopsy may have the ability to find clues of cancer recurrence and metastasis before traditional imaging examinations.

By profiling the genomic alterations in plasma and urine, we detected frequently mutated genes in PCa patients that have been reported in previous studies. Mutations were detected in both plasma and urine samples, suggesting that the liquid biopsy technology, including both plasma- and urine-based NGS tests, may have great potential to impact PCa diagnosis, treatment selection, and disease monitoring. Interestingly, by comparing the mutations detected in plasma and urine samples from the same patients, we found more mutations were detected from urine, but higher mutation frequencies were detected in plasma. These findings suggest that the ctDNA in plasma and urine samples may come from different tumor sources. ctDNA in plasma samples are more likely from metastatic lesions, whereas ctDNA-based alterations in urine are more likely from the primary lesions. This observation suggests different clinical application scenarios of plasma- and urine-based NGS tests.

Although we recruited clinical cases and tried to focus on the evaluation of ctDNA in the blood and urine of PCa patients, there were some limitations in the current study. First, due to the short patients-recruiting period (one year and nine months), we had collected a total of 54 plasma samples and 20 urine samples. Therefore, the sample size seems to be relatively small. Second, samples of plasma and urine were not all paired from patients. Third, the follow-up timeframe was also short (three years and ten months). In the future study, we would continue to collect more samples and to follow up for five years even to ten years. Nevertheless, we thought that ctDNA can be used as biomarkers to increase the sensitivity and specificity of the detection for PCa.

In conclusion, we have used NGS-based liquid biopsy to detect alterations in several genes, including *TP53*, *AR*, *ATM*, *MYC*, and *SPOP* in PCa patients, which may be utilized for monitoring tumorigenesis. Thus, liquid biopsy is a powerful approach for analyzing tumor DNA sourced from blood and urine samples; the latter is the most convenient source for a patient and has great potential for clinical application.

## Data Availability Statement

The data presented in this study can be found in the article/**Supplementary Material**. Requests to access the datasets should be directed to Gang Chen, chgan365@126.com; Guoxiong Xu, guoxiong.xu@fudan.edu.cn.

## Ethics Statement

The studies involving human participants were received and approved by The Ethics Committee of Jinshan Hospital (approval # IEC-2020-S27). Written informed consent was obtained from each participant. The patients/participants provided their written informed consent to participate in this study.

## Author Contributions

Conception and design: GC and GX. Development of methodology: GC, GX, GJ, FC, FX, YZ, CH, YH, and SJ. Acquisition of data (acquired and managed patients, provided facilities, sample collection, etc.): GC, GX, GJ, FC, CH, and JZ. Analysis and interpretation of data (statistical analysis, biostatistics, computational analysis, etc.): GC, GX, FX, YZ, FC, CH, YH, HT, JY, and SJ. Writing-review and editing the manuscript: GC, GX, FX, FC, JY, and SJ. Project administration and study supervision: GC, SJ, and GX. All authors contributed to the article and approved the submitted version.

## Funding

This work was supported by a grant from the Nature Science Foundation of Shanghai (No. 18ZR1405800).

## Conflict of Interest

FX, YZ, YH, and HT are the employees of Huidu Shanghai Medical Sciences Ltd. JY and SJ are the employees and leaders of Huidu Shanghai Medical Sciences Ltd and Predicine. JY and SJ have the stock and other ownership interests of Huidu Shanghai Medical Sciences Ltd and Predicine.

The remaining authors declare that the research was conducted in the absence of any commercial or financial relationships that could be construed as a potential conflict of interest.

## Publisher’s Note

All claims expressed in this article are solely those of the authors and do not necessarily represent those of their affiliated organizations, or those of the publisher, the editors and the reviewers. Any product that may be evaluated in this article, or claim that may be made by its manufacturer, is not guaranteed or endorsed by the publisher.
