# Supplementary material for: Urine- and Blood-Based Molecular Profiling of Human Prostate Cancer
Source: Front Oncol. 2022 Mar 23;12:759791. doi: 10.3389/fonc.2022.759791 (PMC8984469; doi:10.3389/fonc.2022.759791)
Supplement: Supplementary file 1 [file DataSheet_1.pdf]

## Supplementary Figures

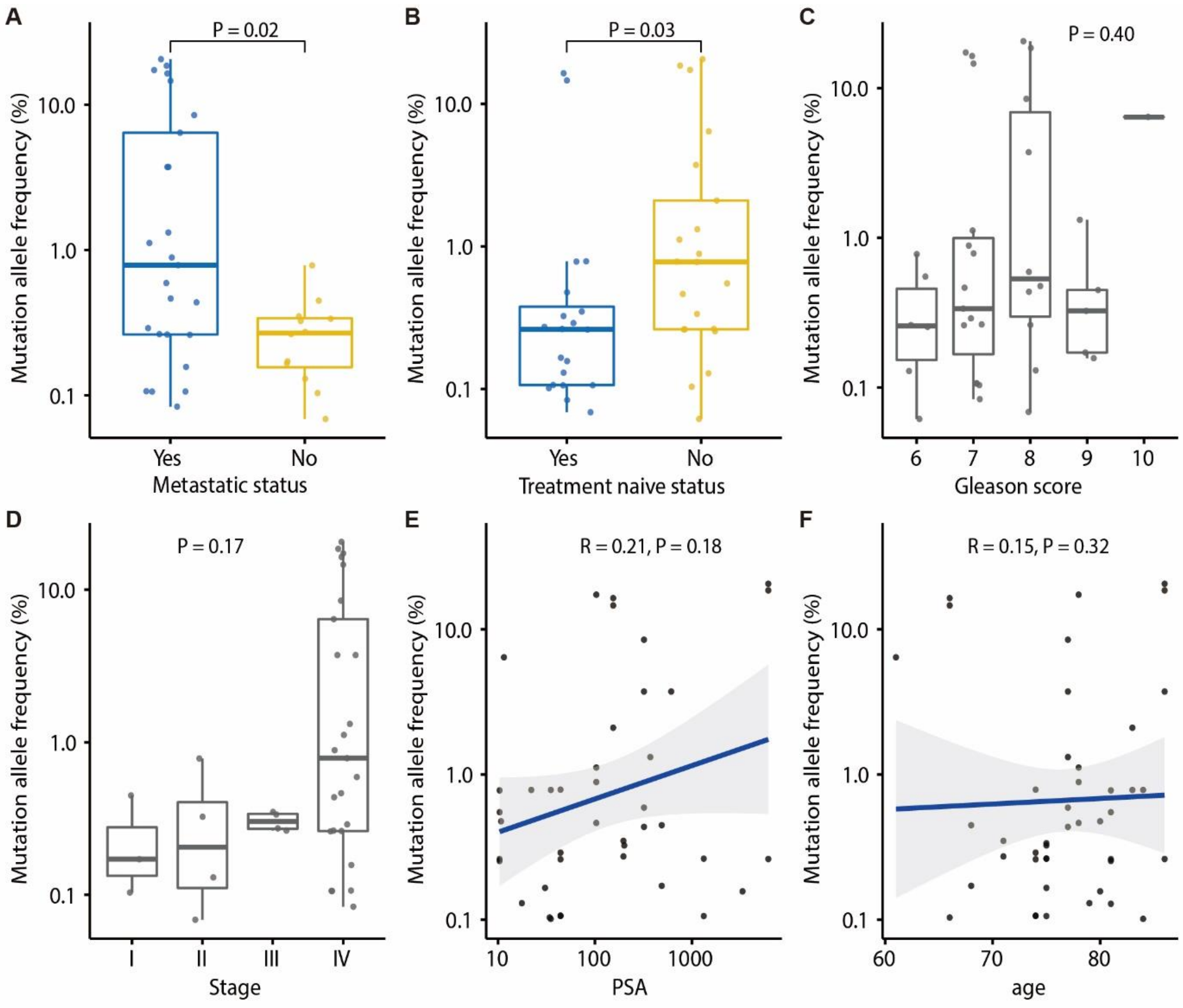


**Supplementary Figure 1 |** Association of mutation allele frequency (MAF) detected in plasma cfDNA and clinical characteristics of prostate cancer. (A) Association of MAF and metastasis status. P-value was calculated by the Mann-Whitney U test. (B) Association of MAF and treatment naïve status. P-value was calculated by the Mann-Whitney U test. (C) Association of MAF and Gleason score. P-value was calculated by the Kruskal-Wallis test. (D) Association of MAF and tumor stage. P-value was calculated by the Kruskal-Wallis test. (E) Association of MAF and PSA. Spearman's rank correlation coefficient and the corresponding P-value are shown. (F) Association of MAF and age. Spearman's rank correlation coefficient and the corresponding P-value are shown. Each dot indicates one sample.

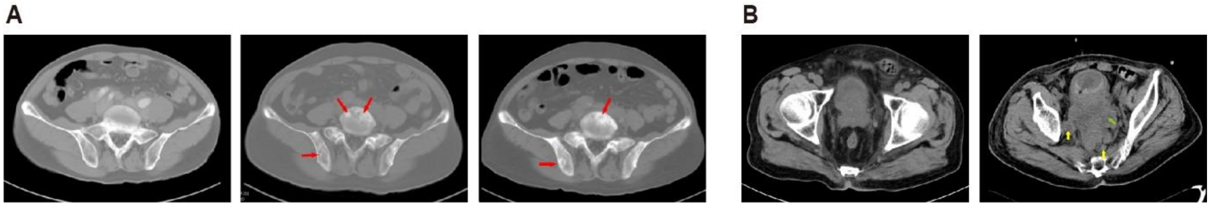


**Supplementary Figure 2 |** Detection of metastasis by computed tomography (CT) scanning. (A) The CT images of the bone metastases in the case 1 patient. At the time of diagnosis, the CT scan did not show bone metastasis (left panel). After oral treatment with bicalutamide and leuprolide for 4 months, the CT scanning indicated multiple bone metastasis (middle panel). After treatment 41 months, the CT scanning still indicated multiple bone metastases (right panel). The red arrows indicate bone metastatic nodules. (B) The CT images of the lymph node metastases in the case 2 patient. At the time of diagnosis, the CT scan did not show metastasis (left panel). After treatment for 25 months, the CT scanning indicated cancer recurrence and pelvic lymph node metastases (left panel). The green arrow indicates the recurrent cancer nodule. The yellow arrows indicate the lymph node metastatic nodules.

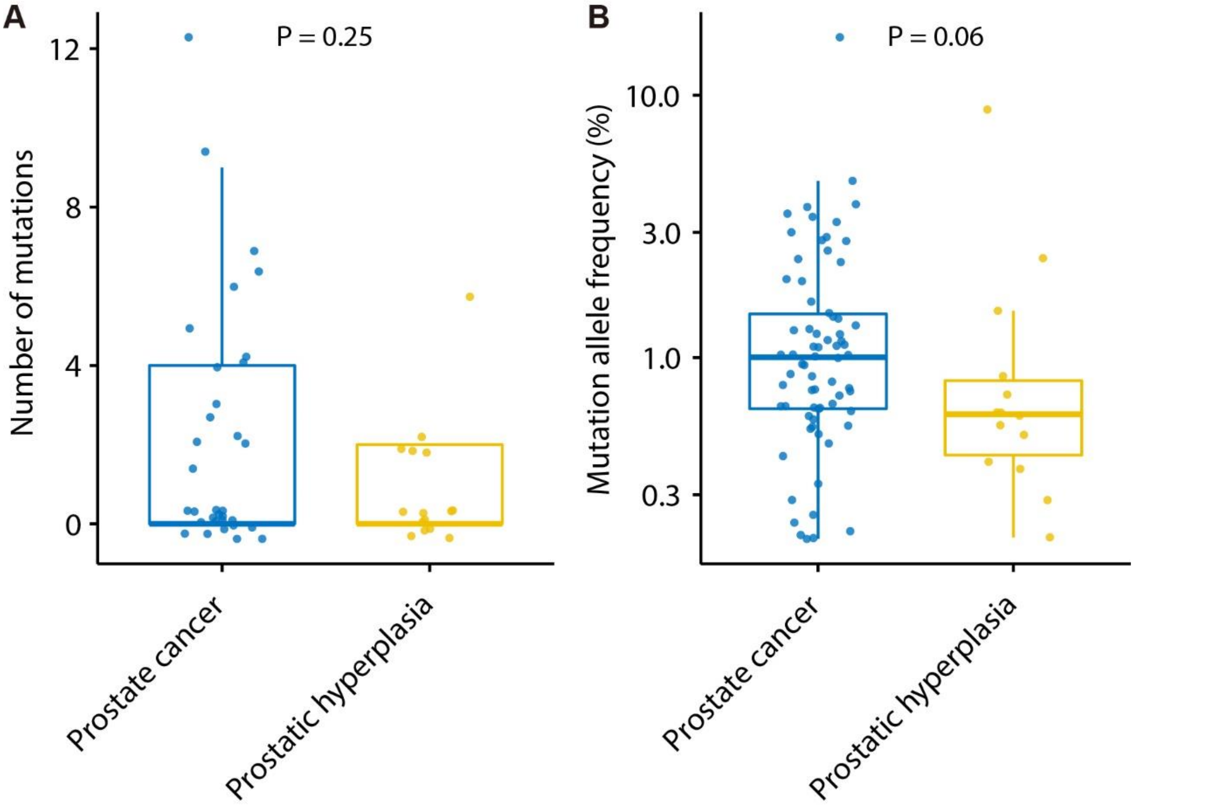


**Supplementary Figure 3 |** Mutation profile of prostate cancer in urine. (A) The number of mutations in prostate cancer and prostatic hyperplasia. (B) Mutation allele frequencies in prostate cancer and hyperplasia. Each dot indicates one sample.

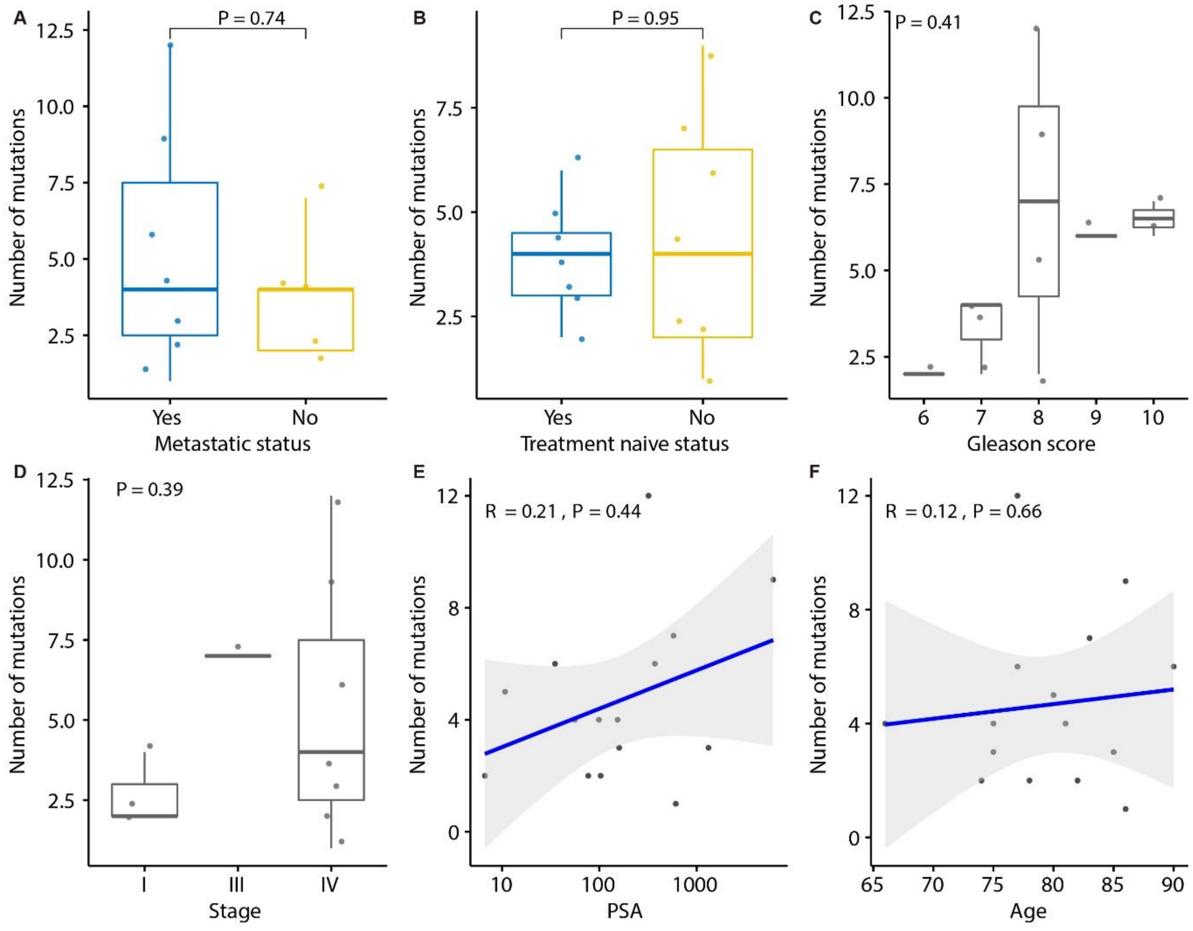


**Supplementary Figure 4 |** Association of the number of mutations detected in urine cfDNA and clinical characteristics of prostate cancer. (A) Association of the number of mutation and metastatic status. P-value was calculated by the Mann-Whitney U test. (B) Association of the number of mutations and treatment naïve status. P-value was calculated by the Mann-Whitney U test. (C) Association of the number of mutations and Gleason score. P-value was calculated by the Kruskal-Wallis test. (D) Association of the number of mutations and tumor stage. P-value was calculated by the Kruskal-Wallis test. (E) Association of the number of mutations and PSA. Spearman's rank correlation coefficient and the corresponding P-value are shown. (F) Association of the number of mutations and age. Spearman's rank correlation coefficient and the corresponding P-value are shown. Each dot indicates one sample.

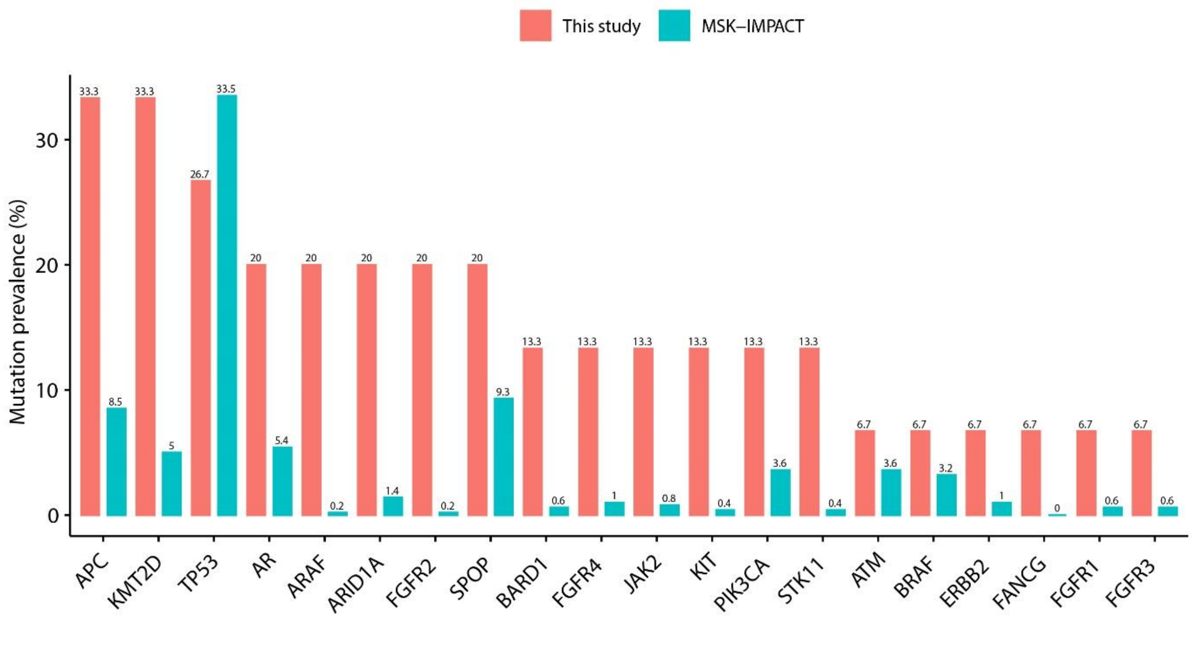


**Supplementary Figure 5 |** Detection of gene mutation prevalence in urine cfDNA samples in this study and tissue samples in MSK-IMPACT Clinical Sequencing Cohort of prostate cancer.
