# Supplementary material for: Urine- and Blood-Based Molecular Profiling of Human Prostate Cancer
Source: Front Oncol. 2022 Mar 23;12:759791. doi: 10.3389/fonc.2022.759791 (PMC8984469; doi:10.3389/fonc.2022.759791)
Supplement: Supplementary file 2 [file DataSheet_2.pdf]

Supplementary Table 1 | Clinical features of patients with BPH and PCa.

Characteristic	**PCa** n=33	**BPH** n=15	***P*-value**
Age (year), median (range)	78 (74.5, 82.5)	71 (65, 78)	**0.0162**
PSA (ng/mL), median (range)	56.00 (26.26, 260.20)	17.60 (8.48, 40.00)	**0.0016**
Smoking status			0.2096
Yes, n (%)	2 (6.1)	1 (6.7)	
No, n (%)	25 (75.8)	14 (93.3)	
Unkown, n (%)	6 (18.2)	0 (0.0)	
Hypertension			0.5240
Yes, n (%)	12 (36.4)	8 (53.3)	
No, n (%)	17 (51.5)	6 (40.0)	
Unkown, n (%)	4 (12.1)	1 (6.7)	
Diabetes Mellitus			0.8476
Yes, n (%)	4 (12.1)	2 (13.3)	
No, n (%)	25 (75.8)	12 (80.0)	
Unkown, n (%)	4 (12.1)	1 (6.7)	
Symptoms			0.1773
LUTS, n (%)	9 (27.3)	9 (60.0)	
Hematuria, n (%)	4 (12.1)	1 (6.7)	
Other symptoms, n (%)	6 (18.2)	0 (0.0)	
Asymptomatic, n (%)	10 (30.3)	4 (26.7)	
Unkown, n (%)	4 (12.1)	1 (6.7)	
Laboratory test			
Hemoglobin			0.1165
Anemia (<120g/L), n (%)	6 (18.2)	0 (0.0)	
Normal, n (%)	22 (66.7)	14 (93.3)	
Unknown, n (%)	5 (15.2)	1 (6.7)	
Creatin			0.3089
High (>137μmol/L), n (%)	3 (9.1)	0 (0.0)	
Normal, n (%)	25 (75.8)	14 (93.3)	
Unknown, n (%)	5 (15.2)	1 (6.7)	
Albumin			0.3760
Low (<40g/L), n (%)	4 (12.1)	4 (26.7)	
Normal, n (%)	24 (72.7)	10 (66.7)	
Unknown, n (%)	5 (15.2)	1 (6.7)	
Fasting blood glucose			0.1909
High (>6.1mmol/L), n (%)	4 (12.1)	5 (33.3)	
Low (<3.9mmol/L), n (%)	0 (0.0)	0 (0.0)	
Normal, n (%)	24 (72.7)	9 (60.0)	
Unknown, n (%)	5 (15.2)	1 (6.7)	
Microscopic hematuria			0.2465
Yes, n (%)	11 (33.3)	2 (13.3)	

No, n (%)	16 (48.5)	11 (73.3)
Unkown, n (%)	6 (18.2)	2 (13.3)

Contingency tables were analyzed using the chi-square test. Numerous data chosen from the normal population were analyzed using Student's *t*-test. Numerous data that were not chosen from the normal population were analyzed using the Mann-Whitney test. BMI, body mass index; BPH, Benign prostatic hyperplasia; LUTS, lower urinary tract symptoms; n, number of cases; PCa, prostate cancer; PSA, prostate-specific antigen.
